# Identification of Human Housekeeping Genes and Tissue-Selective Genes by Microarray Meta-Analysis

**DOI:** 10.1371/journal.pone.0022859

**Published:** 2011-07-27

**Authors:** Cheng-Wei Chang, Wei-Chung Cheng, Chaang-Ray Chen, Wun-Yi Shu, Min-Lung Tsai, Ching-Lung Huang, Ian C. Hsu

**Affiliations:** 1 Department of Biomedical Engineering and Environmental Sciences, National Tsing Hua University, Hsinchu, Taiwan; 2 Institute of Statistics, National Tsing Hua University, Hsinchu, Taiwan; 3 Institute of Athletics, National Taiwan Sport University, Taichung, Taiwan; Kyushu Institute of Technology, Japan

## Abstract

**Background:**

Categorizing protein-encoding transcriptomes of normal tissues into housekeeping genes and tissue-selective genes is a fundamental step toward studies of genetic functions and genetic associations to tissue-specific diseases. Previous studies have been mainly based on a few data sets with limited samples in each tissue, which restrained the representativeness of their identified genes, and resulted in low consensus among them.

**Results:**

This study compiled 1,431 samples in 43 normal human tissues from 104 microarray data sets. We developed a new method to improve gene expression assessment, and showed that more than ten samples are needed to robustly identify the protein-encoding transcriptome of a tissue. We identified 2,064 housekeeping genes and 2,293 tissue-selective genes, and analyzed gene lists by functional enrichment analysis. The housekeeping genes are mainly involved in fundamental cellular functions, and the tissue-selective genes are strikingly related to functions and diseases corresponding to tissue-origin. We also compared agreements and related functions among our housekeeping genes and those of previous studies, and pointed out some reasons for the low consensuses.

**Conclusions:**

The results indicate that sufficient samples have improved the identification of protein-encoding transcriptome of a tissue. Comprehensive meta-analysis has proved the high quality of our identified HK and TS genes. These results could offer a useful resource for future research on functional and genomic features of HK and TS genes.

## Introduction

In the last decade, massive gene expression microarray data has opened an avenue toward transcriptomic study. Benefitting from this progress, the ideas of housekeeping (HK) genes and tissue-selective (TS) genes can now be investigated on a large-scale manner.

Housekeeping genes were first described as those genes always expressed in the cell [1]; the concept was further refined to that HK genes are constitutively expressed to maintain cellular functions [2]. Studies based on large-scale expression data generally identify genes universally expressed in all tissues as HK genes, thus the ubiquitous HK genes are presumed to be candidates for essential genes [3]–[4]. Although some previous studies identified constantly expressed genes among normal and disease tissues as HK genes or reference genes [5]–[7], in this study, we focused on the identification of HK genes which are universally expressed in all normal tissues [2]. Prior to obtaining the commonly expressed HK genes, it is fundamental to identify genes constitutively expressed in each tissue type. When differences in physiology or cell type homogeneity among samples are considered, sufficient numbers of samples are needed to identify the representatively protein-encoding transcriptome (PE transcriptome) of a tissue. Previous microarray-based studies endeavored to collect diverse tissues [3], [8]–[13], but lack of sample availability for each tissue (less than three samples for a tissue in average) has limited the representativeness of the data. Making use of the vast public data by meta-analysis may improve identification of a tissue's PE transcriptome and the follow-up exploration of HK genes.

Tissue-selective genes are predominantly expressed in one or a few biologically relevant tissue types [14]. Tissue selectivity is unlike tissue specificity, which describes genes expressed exclusively in a single tissue type. Thus, a TS gene may also be ubiquitously expressed in many tissue types, while being predominantly expressed in a few. The selective expression of TS genes suggests their possible roles in tissue functions, and therefore they are potential drug targets or disease markers [12], [14]. Besides, correlating genes with tissues in which they are selectively expressed would facilitate future functional investigations. Methods for the identification of TS genes are frequently investigated and improved [9], [14]–[19]. In addition to the method applied, diversity of tissue types is decisive for assigning TS genes. The selective expression of a gene would be correctly recognized by contrast between relevant and irrelevant tissues. Compiling samples from public data sets provides a way to collect as many varied samples as possible. A recent study which developed a computational approach for combining heterogeneous microarray expression profiles has emphasized this idea [19].

These two transcriptomic extremities, HK genes and TS genes, have prompted research interest in their expressional and functional characteristics [8]–[9], as well as their genomic structural [10], [20]–[22], evolutionary [23]–[24], and epigenetic features [13]. However, low consensus among HK gene lists of these studies were reported by recent studies [11]–[12], which may result in discordant conclusions about features of HK and TS genes. For example, HK genes were described as shorter in genomic structure when compared to TS genes [10], [20], but were stated as less compact by a recent study [23]. Accurately selected HK and TS genes should be the foundations for investigating their genomic features. Thus, representative HK and TS genes for characterizing their functional and genomic features are desired.

In this study, we compiled 1,431 quality controlled samples in 43 normal human tissue types from M^2^DB, the microarray meta-analysis database [25], which encompasses more than 10,000 human-curated samples from published Affymetrix GeneChips. To define the PE transcriptome of each tissue, we developed the fraction Present weighted expression intensity (FPEI), which overall performs better than expression intensity and fraction Present [26] in gene expression assessment. We also show that at least ten samples are required to robustly identify the PE transcriptome of a tissue, which reveals the feasibility and the power of meta-analysis in exploring HK and TS genes. The FPEI developed in this study could be easily applied to the data analysis of other microarray platforms, since most of platforms provide expression intensity and quantitative detection call. With the FPEI defined tissue PE transcriptomes, this study identifies 2,064 HK genes and 2,293 TS genes for 43 tissue types. By applying functional enrichment analysis, our defined HK genes are mainly involved in fundamental cellular functions, and TS genes are closely related to tissue-relevant functions and diseases. Although agreements among existing HK gene lists are only moderate (the consensuses among them range from 10% to 80%), most HK gene lists enrich functions similar to those enriched by our HK genes. Results also show that the TS genes of specific blood cells are still functionally relevant to their tissue-origin, which sheds light on future fine-tuning of samplings in improving gene selectivity identification.

## Results

### Representative protein-encoding transcriptomes

Most previous HK gene studies identified expressed genes in each tissue type by applying a threshold to expression intensity. Recently, filtering by *fraction of Present detection calls* (fraction Present) of samples in a group has shown to preserve reliably detected and biologically informative probe sets [26]–[27], which inspired us to use fraction Present as an alternative indicator. In addition, this study further developed the fraction Present weighted expression intensity (FPEI) as a novel indicator to assess gene expression in a tissue.

**Figure 1:**
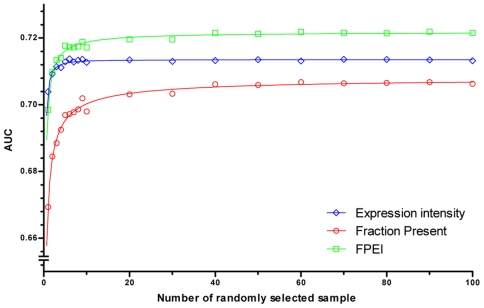
Performance of the indicators and the effect of sample size. Area under the ROC curve (AUC) is used to evaluate the performance of assessing gene expression (See details in Text S1). The AUCs for FPEI are significantly larger than that for expression intensity with more than five randomly selected samples (*P*<2.9×10^−11^ by paired *t*-test). The fitted lines are plotted by the Hill function *AUC* = *aN^b^*/(*c^b^*+*N^b^*), where *AUC* is the area under the ROC curve and *N* is the number of randomly selected samples. Error bars are not shown for clarity.

To evaluate the three indicators and the sample size in assessing gene expression in a tissue, our study used muscle samples as an example to perform the receiver operative characteristic (ROC) analysis (Text S1). For all of the three indicators, results of ROC analysis indicated that more than ten samples were needed to reach a robust identification of expressed genes for muscle (Figure 1). Using expression intensity as an indicator requires fewer samples than the others did to reach a performance plateau; however, further increasing the sample size did not improve its performance. By contrast, sufficient samples were needed to improve the discriminability of fraction Present, which limited the application of fraction Present in tissues with few samples. The FPEI outperformed the others in the ROC analysis, due to compensating for the high noise nature of low expression intensity, and preserving the discriminability of high expression intensity (Text S1). Besides, fraction Present was positively correlated to expression intensity, especially for selectively expressed genes (Figure S1). Thus, weighting by fraction Present further emphasized the selective pattern of expression intensity, which made FPEI superior for assigning TS genes. Overall, the ROC analyses and the selectivity-discriminability of FPEI indicated that FPEI was better than expression intensity and fraction Present in assessing gene expression and reflecting selective expression. Thus, we used FPEI as the indicator, and FPEI-identified expressed genes in each tissue as the representative PE transcriptomes for the follow-up HK and TS genes exploration.

### Housekeeping genes and functions

Adapting FPEI for searching representative PE transcriptomes, we identified 2,064 genes universally expressed across all analyzed 43 tissues as HK genes (16.4% of total analyzed genes; HK genes and their FPEI were listed in Table S1). By applying functional enrichment analysis with Gene Ontology [28] (GO) and KEGG pathway [29], we found that these 2,064 HK genes enrich functions of fundamental biological processes such as translation, RNA metabolic process, ribosome biogenesis, oxidative phosphorylation, proteolysis and its regulation, molecular transport, and cell cycle (Table 1; full functional enrichment results were shown in Table S2). As many as one-fourth (27%) of the HK genes were related to gene expression (which included the functions concerning translation, RNA metabolic process, and ribosome biogenesis). In addition, 21% of the HK genes were associated with molecular transport. Approximately 80% of the HK genes were involved in the enriched functions (Table 1), and most of the remaining 20% HK genes were not categorized by GO biological process nor KEGG pathway (328 out of 413). This indicated that our defined HK genes were highly focused on these fundamental functions. Genes associated with ribosomal small subunit biogenesis and translational elongation were almost ten times more highly expressed than average HK genes (Table 1), which were mentioned as the most abundantly expressed among HK genes in previous studies [3], [9], [12]. Genes in the tRNA metabolic process function were the least expressed among our HK genes (Table 1). They are generally known as universally expressed for translational need; however, they were not enriched in previous HK gene lists (see below).

**Table 1:** Functional enrichment analysis of HK genes.

Function	Gene No.	% HK gene^a^	Average FPEI^b^
**Translation**	**186**	**9.0**	
GO:0006413∼translational initiation	27	1.3	1,298.2
GO:0006414∼translational elongation	86	4.2	11,960.0
GO:0006417∼regulation of translation	43	2.1	995.1
**RNA metabolic process**	**275**	**13.3**	
GO:0006397∼mRNA processing	128	6.2	740.7
GO:0006399∼tRNA metabolic process	34	1.6	567.5
GO:0000245∼spliceosome assembly	18	0.9	665.7
**Ribosome biogenesis**	**108**	**5.2**	
GO:0022613∼ribonucleoprotein complex biogenesis	75	3.6	786.0
GO:0006364∼rRNA processing	38	1.8	944.7
GO:0042274∼ribosomal small subunit biogenesis	11	0.5	12,263.0
**Oxidative phosphorylation**	**211**	**10.2**	
GO:0006119∼oxidative phosphorylation	53	2.6	1,912.1
GO:0022900∼electron transport chain	50	2.4	1,466.1
**Proteolysis**	**201**	**9.7**	
GO:0006508∼proteolysis	193	9.4	799.0
GO:0016567∼protein ubiquitination	37	1.8	818.0
**Regulation of Protein metabolic process**	**160**	**7.8**	
GO:0031398∼positive regulation of protein ubiquitination	43	2.1	1,220.0
GO:0051248∼negative regulation of protein metabolic process	67	3.2	1,082.1
**Transport**	**432**	**20.9**	
GO:0015031∼protein transport	180	8.7	813.9
GO:0048193∼Golgi vesicle transport	45	2.2	747.4
GO:0051169∼nuclear transport	47	2.3	777.7
**Cell cycle**	**159**	**7.7**	
**Other**	**223**	**10.8**	
GO:0006457∼protein folding	50	2.4	1,001.0
GO:0045454∼cell redox homeostasis	24	1.2	1,155.2
GO:0010608∼posttranscriptional regulation of gene expression	62	3.0	897.9
GO:0044419∼interspecies interaction between organisms	78	3.8	1,033.6
**Unknown** c	**328**	**15.9**	
**Total genes in enriched functions**	**1,651**	**80.0**	

Enriched functions were consolidated into 8 functional groups (bold terms). Only representative GO terms were listed under each functional group. Full results were provided in Table S2.

a Percentage of 2,064 HK genes related to the function.

b The mean of FPEI for genes in the function.

c Genes not categorized by GO biological process nor KEGG pathway.

**Figure 2:**
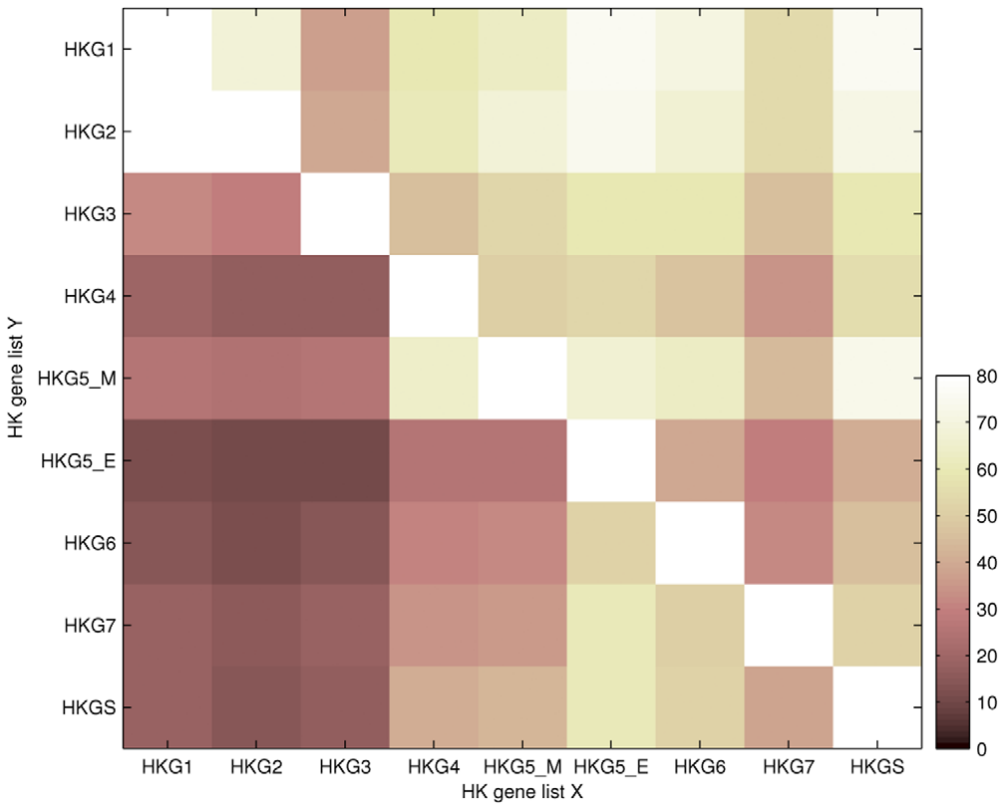
Agreement of HK gene lists. This graph shows the concordance of genes for pairs of HK gene lists presented in Table 2. The color represents the percentage of genes in the HK gene list Y that are also identified as HK genes in the HK gene list X. Overall values in a column represents that whether the HK gene list X agree with others, while overall values in a row represents that whether the HK gene list Y is approved by others. The lower are the overall row values, the more genes are unique to the HK gene list Y.

We compared our study with seven microarray-based studies (referred to as gene lists HKG1-7 in Table 2) and one EST-based meta-analysis (referred to as gene list HKG5_E in Table 2). Although the three pioneering HK gene lists identified about 500 genes as HK genes (HKG1-3), recent studies have presented about 2000 HK genes on average (HKG4-7 and HKGS in this study), which roughly corresponds to 13% of total human genes. In general, agreements among HK gene lists were moderate (Figure 2). Small sizes of HKG1-3 resulted in their low agreements with others (overall dimmer columns of HKG1-3, Figure 2). However, HKG1-2 have been highly approved by recent studies (rows HKG1-2), which implied that they were in consensus of recently identified HK genes. The HKG5_E of the EST-based study agreed well with others (column HKG5_E). However, we found that as many as 1,034 genes are unique to HKG5_E, resulting in a substantial percentage not approved by others (overall dimmer row of HKG5_E). HKG7 discernibly disagreed with other recent studies (overall dimmer column of HKG7), even though it was closed to the average size of recent HK gene lists. By comparing results of functional enrichment analysis (Figure S2), we found that enriched functions common to most HK gene lists were in agreement with functional groups listed in Table 1. That is, all HK gene lists commonly enriched functions concerning translation, RNA metabolic process, ribosome biogenesis, proteolysis and its regulation, molecular transport, and cell cycle. Besides, all HK gene lists except HKG5_E showed enrichment in oxidative phosphorylation. The overall dimmer functional enrichment patterns of HKG3 and HKG7 reflected their disagreement with others (Figure S2). This disagreement was also reflected in Figure 2 as we mentioned above. Our HK gene list showed a highly significant enrichment of these common functions when compared to other lists (overall lighter column of HKGS, Figure S2). This corresponded to the fact that our HK genes were highly focused on these fundamental functions (Table 1). Enriched functions unique to our HK gene list concerned tRNA metabolic process, proteolysis, and molecular transport (Figure S3). These were in the category of common HK functions.

**Table 2:** Comparison of HK gene studies.

Index	Study	Sample No.	Tissue No.	HK gene No.	Platform
HKG1	Warrington et al. (2000) [8]	120	8	535	Affymetrix, HuGeneFL
HKG2	Hsiao et al. (2001) [9]	59	19	451	Affymetrix, HuGeneFL
HKG3	Eisenberg et al. (2003) [10]	46	32	575	Affymetrix, HG-U95A from [40]
HKG4	Tu et al. (2006) [3]	142	79	1,789	Affymetrix, HG-U133A and GNF1H from [41]
HKG5_M	Zhu et al. (2008) [11]	18	18	1,260	Affymetrix, HG-U133A and GNF1H from [41]
HKG5_E	Zhu et al. (2008) [11]	2,502^a^	18	3,140	EST data from UCSC annotation database [54]
HKG6	Dezso et al. (2008) [12]	31	31	2,374	Applied Biosystems P/N4337467
HKG7	She et al. (2009) [13]	42	42	1,522	Custom two-color high-density microarray
HKGS	This study	1,431	43	2,064	Affymetrix, HG-U133A and HG-U133-Plus2 from M^2^DB [25]

EST, expression sequence tag.

a Number of cDNA libraries used for EST study.

### Tissue-selective genes and functions

In this study, we adapted the tissue-selective score developed in a previous study [18] to identify 2,293 TS genes (18.3% of total analyzed genes; TS genes and their FPEI were listed in Table S3). The TS genes had tissue-selective scores higher than the random permutation-defined threshold, and were expressed in at least one tissue. The FPEI of TS genes accurately reflected the specialized role of genes in corresponding tissues (Table S3). For example, the troponin family genes *TNNC1* and *TNNI1* were commonly expressed in heart and muscle; *TNNI3* and *TNNT2* were uniquely expressed in heart; *TNNC2*, *TNNI2*, *TNNT1*, and *TNNT3* were uniquely expressed in muscle. The myosin family genes *MYL2* and *MYL3* were common to both tissues; *MYH7B* and *MYL7* were unique to heart, while *MYH1*, *MYH2*, *MYH4*, *MYH8*, and *MYL1* were unique to muscle. These results were consistent with conventional knowledge of their functions, except that *MYL2* was well known to be associated to heart contraction and cardiomyopathy [30]. Our data showed an extremely high expression level of *MYL2* in the muscle (Table S3), which may provide new insight into role of *MYL2* in muscle contraction.

**Figure 3:**
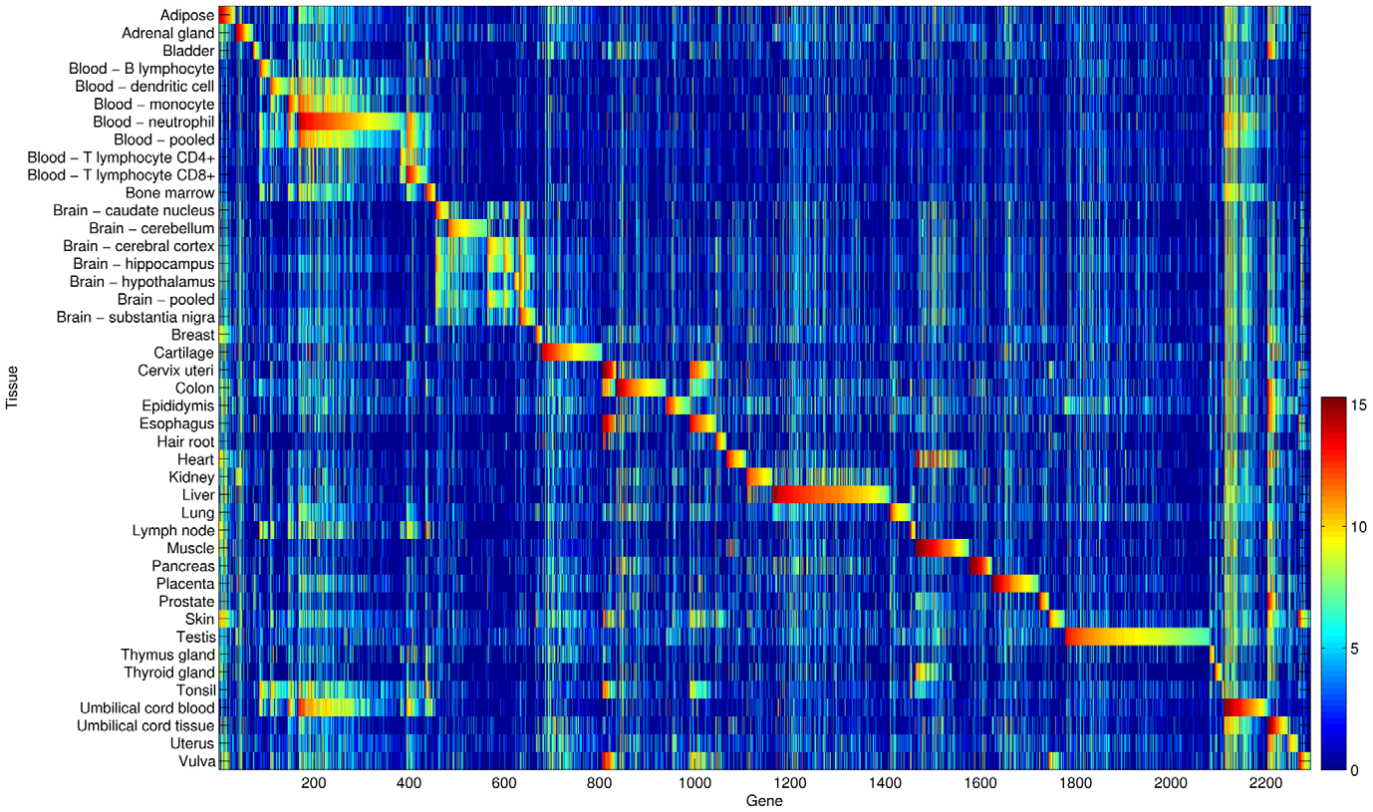
Expression patterns of 2,293 TS genes. Genes are sorted according to tissue name and then FPEI value. The associations between TS genes with their corresponding tissue and other tissues can be easily visualized.

As shown by the expression pattern of TS genes (Figure 3), genes selectively expressed in blood, brain, liver, and testis corresponded to 15.2%, 9.2%, 10.7%, and 13.3% of TS genes, and dominated the number of total TS genes. Pancreas and testis TS genes were seldom expressed in other tissues, which indicated that their functions were specialized for these tissues. The cervix and esophagus exhibited extremely similar gene expression patterns; both of their TS genes were related to epidermis development (Table S4). The tonsil showed a combined gene expression pattern of epidermis and immune tissues, which implied that tonsil samples comprised both tissues. However, the composition of two tissue types lowered the selective expression pattern, which resulted in fewer TS genes assigned to the tonsil. Tissues can be grouped according to functional similarities by applying a hierarchical clustering analysis to expression patterns of TS genes (Figure S4).

This study also examined the enriched functions of TS genes with GO and KEGG pathway, and the related diseases with annotation of the Genetic Associated Database [31]. In general, our defined TS genes were highly related to tissue-corresponding functions and diseases (Table S4). The results of a specific subtype of blood cells were also associated to their roles, such as the B cell receptor signaling function of the B lymphocyte, the regulation role of CD4+ T lymphocyte, the cytotoxicity role of CD8+ T lymphocyte, and the correlation between multiple sclerosis to monocytes and neutrophils (Table 3). Though brain subtypes were mostly defined by histological components of the brain rather than cell types, some of their TS genes were still highly related to corresponding functions and diseases. For example, cerebellum selectively expressed genes were associated with epilepsy; caudate nucleus selectively expressed genes were related to cognition; and hypothalamus selectively expressed genes were associated with behavior (Table 3). Overall, our defined TS genes excellently reflected their tissue-origin, and provided a useful resource to facilitate the investigation and annotation of genes to their corresponding functions.

**Table 3:** Functional enrichment analysis of TS genes for blood and brain subtypes.

Tissue	Gene Ontology biological process	KEGG pathway	Genetic association database
Blood - B lymphocyte	GO:0002376∼immune system process	hsa04662:B cell receptor signaling pathway	
Blood - monocyte	GO:0006955∼immune response	hsa04640:Hematopoietic cell lineage	multiple sclerosis
Blood - neutrophil	GO:0002376∼immune system process	hsa04060:Cytokine-cytokine receptor interaction; hsa04144:Endocytosis	malaria; multiple sclerosis
Blood - T lymphocyte CD4+	GO:0002682∼regulation of immune system process; GO:0050863∼regulation of T cell activation	hsa04660:T cell receptor signaling pathway	diabetes, type 1; celiac disease; myasthenia gravis
Blood - T lymphocyte CD8+	GO:0002376∼immune system process; GO:0031343∼positive regulation of cell killing	hsa04650:Natural killer cell mediated cytotoxicity	Wegener's granulomatosis
Brain - caudate nucleus	GO:0050877∼neurological system process; GO:0050890∼cognition	hsa04020:Calcium signaling pathway	alcohol abuse
Brain - cerebellum	GO:0007154∼cell communication	hsa04742:Taste transduction	epilepsy
Brain - hypothalamus	GO:0007267∼cell-cell signaling; GO:0007610∼behavior		

Only portion of the blood and brain subtypes were listed and only representative functions were shown for listed subtypes. Full results for all tissue types were provided in Table S4.

## Discussion

With the introducing of FPEI, we defined representative transcriptomes of 43 tissue types, and identified reliable and biologically informative HK and TS genes.

For meta-analysis of HK and TS genes, we consider sample size, tissue type, quality control of the data, and selection criteria for identifying expressed genes as important factors for obtaining reliable results. A number of previous studies have developed approaches to estimate required sample size to identify differentially expressed genes across experimental variables [32]–[34]. According to considerations such as experimental design, multiple testing, or the utilized pilot data, the required sample size may range from tens to hundreds to reach a sufficient statistic power. For example, by estimating the standard deviation of each gene from a permutation-based analysis with a set of pilot data, Tibshirani [33] showed that about 100 samples are needed to reach a false discover rate of 5%. These studies elucidate the variability of gene expression and the need of sufficient samples to assess differential gene expression. We show that at least ten samples are needed for a histologically uniform tissue, like muscle, to reach a robust gene expression assessment (Figure 1). For organs comprising promiscuous cell or tissue types, variances are expected from sampling to sampling, thus more samples are recommended to assess their PE transcriptome. A previous study examined the effects of biological variability to sample size further emphasized this idea [35].

Tissue types included in the study may impact HK and TS gene exploration, as a lack of representative tissue types with distinct functions may misidentify some genes as either HK genes or TS genes. Moreover, in the detail sub-typing study of blood, we show that the delicate sub-typing of immunocytes has precisely assigned the immune TS genes to their functionally related cell types (Table 3). This result may shed light on delicate investigation of selective gene expression when the pure cell sub-typing of a tissue is technically available. On the other hand, although the sub-typing of brains is based on histological components rather than cell types, some of the TS genes are well related to the corresponding functions of their histological components. These two results suggested that selective expression of genes might be under the regulation of the microenvironment of functional component of a tissue or organ as well as originate from cell type differentiation. Follow-up studies in poorly sampled or sub-typed tissues may be invaluable for supplementing the understanding of gene expression in normal human tissues.

The availability of raw data is essential for meta-analysis; using pre-processed data by different algorithms will introduce variations into the results [36]–[37]. And quality control of samples is important; inclusion of low quality samples may lead to variance of the data [25], which could blur subsequent biological investigations. Previously MicroArray Quality Control (MAQC) consortium has shown that good laboratory proficiency and improved data quality significantly enhances inter-laboratory and inter-platform reproducibility [38]. Besides, accurate categorization and annotation of samples is also important. The data source of this study, M^2^DB, has curated the annotation of samples and categorized them with controlled vocabularies [25]. Moreover, users can easily exclude incompatible samples by employing quality control methods offered by the database. These methods ensure the quality of our used data and the accuracy of annotation.

Methods for the identification of genes predominantly expressed in a few tissues have been proposed in many studies addressing the exploration of TS genes [9], [14]–[19]. Among these methods, a single tissue-specificity index developed by a previous study [17] was relatively simple and efficient, and was frequently applied and improved by follow-up studies [14], [18], [39]. In this study, we applied the improved tissue-selective score [18] with FPEI to identify genes that are tissue-selectively expressed. Instead of using FPEI, if we followed the same procedures using expression intensity as the indicator, only half of the TS genes could be identified and many tissues would not be linked to related functions with these genes. Our results dealing with TS genes prove the effectiveness of our method.

The Gene Expression Atlas [40]–[41] greatly facilitates the investigation of PE transcriptomes of human tissues and follow-up research regarding HK and TS genes [3], [10]–[11], [20], [22], [39], [42]–[43]. As suggested by the authors, these studies used global median of expression intensity (the scaling target of the MAS5 normalized data) as the cutoff value to define expressed genes. However, we find that applying the global median as the cutoff to expression intensity tends to set 50% of genes as expressed for all tissues (Figure S5). We suggest that establishing a universal cutoff for expression intensity is too rigid to reflect biological variety of gene expression. By applying a threshold of 100 to FPEI, the percentage of expressed genes in each tissue ranges from 36% (thyroid gland) to 55% (epididymis) (Figure S5), which is not far off the averaged percent present of all 1431 samples (41%). From these results, we can see that FPEI could shape the variety of nature in gene expression.

Through comparing the microarray data from Gene Expression Atlas II [41] with meta-analysis of public EST data in exploring HK genes [11], it was reported that microarray data results in a significant underestimation of the number of HK genes. We finds an overall lower percent present (the percentage of Present detection calls provided by MAS5 algorithm for a sample) in the samples in Gene Expression Atlas II (28%) when compared to samples from other experiments (42%) (Figure S6). This may indicate that the quality of microarray data from Gene Expression Atlas II is incompatible with data from other experiments, and elucidates that underestimation of gene expression is not a general characteristic of microarray data. In the EST-based study, the authors identified a transcript as expressed for a tissue once the transcript is reliably detected in any EST library [11]. This loose selection criterion may result in high false positives and overestimation of HK genes for the HKG5_E, as pointed out in Housekeeping genes and functions sub-section of Results.

Although only a moderate degree of agreements exists among HK gene lists (Figure 2), according to functional enrichment analysis, most HK gene lists commonly enrich several fundamental functions (Figure S2). These functions are in agreement with functional groups generated from our HK gene list (Table 1). Thus, Table 1 represents the major functional categories of HK genes, and provides guidelines for future investigations into HK genes.

There are seven genes, which are identified as TS genes in our study as well as universally expressed HK genes (Figure S7), and five of them are selectively expressed in umbilical cord blood. These five genes were also frequently identified as HK genes in previous studies. The expressional duality of these five genes caused us to pose an assumption about their hematopoietic potency. Among them, *MKRN1* was recently correlated to the telomerase elimination pathway [44], *ADIPOR1* was identified as a novel growth factor for hematopoietic stem cells [45], and *BNIP3L* was reported in regulation of erythrocyte maturation [46]. Thus, we speculate on the hematopoietic roles of *RNF10* and *MARCH8*, the two ring finger proteins whose functions are not yet understood well, to be proven in the future research.

## Materials and Methods

### Data collection and pre-processing

We compiled 1,431 raw files of Affymetrix GeneChip HG-U133A or HG-U133-Plus2 in 43 normal human tissues from 104 publicly available data sets through our recently published database [25]. Samples in the database are human-curated and consolidated into corresponding tissue or organ types according to annotation from ArrayExpress [47] and the original papers. To investigate delicate functions of the blood and brain with presently available sampling depths, we classified blood and brain samples into representative and non-redundant subtypes. Blood samples representing a specific cell type were separated into blood subtypes and otherwise collected into the blood-pooled class. Brain samples representing a specialized histological component were separated into brain subtypes and otherwise collected into the brain-pooled class. Samples classified as part of the brain cortex according to Brodmann's area were grouped into the brain-cerebral cortex class.

This study validated sample quality by examining five *simpleaffy* [48] generated quality control factors with a quality assessment method PMVO [49] (Parametric Multivariate Outlier labeling) in R/BioConductor [50]. Five quality control factors were scaling factor, percent present calls provided by the MAS5 algorithm [51], average background, and 3′/5′ ratios of *ACTB* and *GAPDH*. We then excluded samples identified as outliers by PMVO with an overall error rate of 0.01, and excluded tissues with less than five samples. After that, 1,431 samples in 43 normal tissues or tissue subtypes were of compatible quality and used for follow-up analysis (detailed sample information are provided in Table S5).

All samples were uniformly processed by MAS5 algorithm in R/BioConductor. Only 22,277 probe sets common to both HG-U133A and HG-U133-Plus2 were used. We re-scaled 30%-trimmed mean intensity of these 22,277 probe sets of each sample to 200, to make the intensities of probe sets from two array generations comparable. Detection calls of 1,431 samples showed that about 41% of probe sets were present.

We used the annotation build 30 of NetAffx [52] from Affymetrix official website to identify probe sets. Quality of probe sets were validated as follows: 1) probe sets with transcripts matching annotations other than *Matching probes* (Grade A) were excluded to ensure reliable annotation matches; and, 2) probe sets with multiple Entrez Gene IDs were excluded. Finally, we obtained 19,106 reliable probe sets, which corresponded to 12,559 unique Entrez Gene IDs. For each tissue, expression intensities and present calls from probe sets of a gene were summarized into expression intensity and fraction Present for the gene, respectively.

### Selection of housekeeping genes

In addition to expression intensity and fraction Present, we weighted expression intensity with fraction Present for each gene in each tissue type to simply integrate the two indicators:
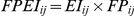
where *FPEI_ij_* is the fraction Present weighted expression intensity (FPEI) for gene *i* in tissue type *j*, which combines the expression abundance *EI_ij_* and the confidence of detection *FP_ij_*. We considered a gene constitutively expressed in a tissue type if its *FPEI_ij_* exceeded 100, the product of commonly applied expression intensity of 200, and a relatively fair fraction Present of 50%. By applying the threshold to *FPEI_ij_*, housekeeping genes were defined as with *FPEI_ij_* above 100 in all 43 studied tissues.

### Selection of tissue-selective genes

We computed a tissue-selectivity score [18] based on the *FPEI_ij_* according to
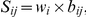
where *b_ij_* is the normalized *FPEI_ij_*

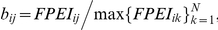
and *w_i_* is the gene-specific weight defined as tissue-specificity index [17] across *N* tissues
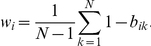
The significance threshold for the tissue selectivity score was defined by performing a permutation test. Gene and tissue pairs in the original data were randomly permutated to obtain 10,000 pseudo samples. Tissue-selectivity scores were then calculated with these samples to select a cutoff value above which are 1% of the random scores. Finally, genes with the original tissue-selectivity score *S_ij_* above the cutoff and with *FPEI_ij_* above 100 in at least one tissue (that is, considered as expressed in at least one tissue) were considered as tissue-selective genes.

### Functional enrichment analysis of HK genes and TS genes

Functional enrichment analysis were accessed through DAVID [53], which performed a modified Fisher's exact test (with EASE score as the *P*-value) to select over-represented functions. We applied the biological process category of GO and the KEGG pathway for functional enrichment analysis of HK genes. Functions were considered as significant with FDR-adjusted EASE scores smaller than 0.05, and were consolidated into eight functional clusters according to DAVID functional annotation clustering and GO term lineage. For TS genes, in addition to GO and KEGG pathway, we applied Genetic Associated Database to find the associated diseases, by which functions or diseases with EASE scores smaller than 0.01 were considered as significant.

## Supporting Information

Figure S1 **Expression intensity and fraction Present of four example genes.** Two HK genes *ACTB* and *GAPDH*, and CD8+ T lymphocyte selective gene *CD8B* and liver selective gene *CYP4A11* are shown as examples. Weighting expression intensity with fraction Present would emphasize the selective expression of *CD8B* in T cell related tissues, especially in CD8+ T lymphocyte, as well as the selective expression of *CYP4A11* in liver and kidney.
(PDF)

Figure S2 **Comparison of enriched functions common to HK gene lists.** This graph shows enriched functions common to most HK gene lists presented in Table 2. Functions enriched in at least eight out of nine HK lists are shown. The color reflects the negative logarithm-transformed FDR-adjusted EASE scores (see [53]).
(PDF)

Figure S3 **Comparison of enriched functions unique to each HK gene list.** This graph shows enriched functions unique to each HK gene list presented in Table 2. The color reflects the negative logarithm-transformed FDR-adjusted EASE scores (see [53]).
(PDF)

Figure S4 **Clustering of 43 tissues with expression pattern of TS genes.** Tissues were hierarchically clustered with average linkage according to gene expression patterns (shown in Figure 3). Tissues are grouped with functional similarities.
(PDF)

Figure S5 **Percentage of expressed genes in each tissue with three different indicators.** Percentages of expressed genes range from 36% (thyroid gland) to 55% (epididymis) by applying a cutoff of 100 to FPEI (green bars). These numbers are proportional to that identified by cutoff of 50% to fraction Present (red bars) and the mean percent presents of each tissue. Applying a cutoff of 200 (the scaling target of the MAS5 normalized data) to expression intensity tends to set 50% of genes as expressed (blue bars). Mean percent presents are not shown for clarity.
(PDF)

Figure S6 **Comparison of percent present of samples in Gene Expression Atlas II to other experiments.** Samples of Gene Expression Atlas II show a significant lower percent present when compared to other samples used in this study (*P* = 1.8×10^−64^ by two-tailed *t*-test). Bars are means from different sample origins; error bars show s.d..
(PDF)

Figure S7 **Expression of seven both housekeeping and tissue-selective genes.** *FDX1* is selectively expressed in adrenal gland (2), and *TUBA3C* is selectively expressed in testis (36). Other five genes, *BNIP3L*, *RNF10*, *MKRN1*, *ADIPOR1*, and *MARCH8*, are selectively expressed in umbilical cord blood (40).
(PDF)

Table S1 **The list of 2064 HK genes and their FPEI in 43 tissues.**
(XLS)

Table S2 **Functional enrichment analysis results of HK genes.**
(XLS)

Table S3 **The list of 2293 TS genes and their FPEI in 43 tissues.**
(XLS)

Table S4 **Functional enrichment analysis results of TS genes.**
(XLS)

Table S5 **The list of all 1431 samples.**
(XLS)

Text S1 **Results for Receiver Operating Characteristic (ROC) analysis with 93 muscle samples.**
(PDF)
