# Supplementary material for: Identification of Human Housekeeping Genes and Tissue-Selective Genes by Microarray Meta-Analysis
Source: PLoS One. 2011 Jul 27;6(7):e22859. doi: 10.1371/journal.pone.0022859 (PMC3144958; doi:10.1371/journal.pone.0022859)
Supplement: Figure S1 — Expression intensity and fraction Present of four example genes. Two HK genes ACTB and GAPDH, and CD8+ T lymphocyte selective gene CD8B and liver selective gene CYP4A11 are shown as examples. Weighting expression intensity with fraction Present would emphasize the selective expression of CD8B in T cell related tissues, especially in CD8+ T lymphocyte, as well as the selective expression of CYP4A11 in liver and kidney. (PDF) [file pone.0022859.s001.pdf]

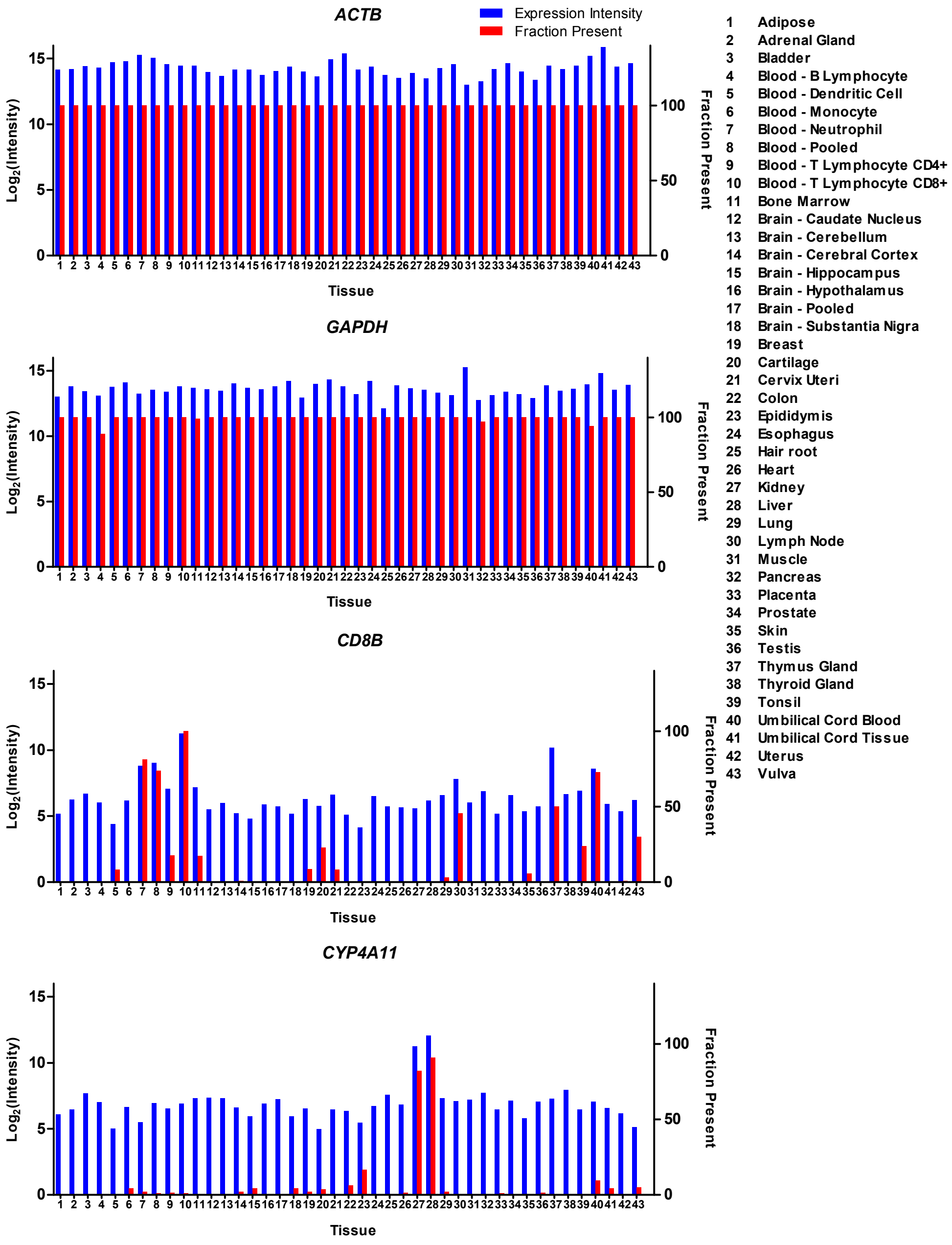

Figure S1

Expression intensity and fraction Present of four example genes. Two HK genes *ACTB* and *GAPDH*, and CD8+ T lymphocyte selective gene *CD8B* and liver selective gene *CYP4A11* are shown as examples. Weighting expression intensity with fraction Present would emphasize the selective expression of *CD8B* in T cell related tissues, especially in CD8+ T lymphocyte, as well as the selective expression of *CYP4A11* in liver and kidney.
