# Supplementary material for: Identification of Human Housekeeping Genes and Tissue-Selective Genes by Microarray Meta-Analysis
Source: PLoS One. 2011 Jul 27;6(7):e22859. doi: 10.1371/journal.pone.0022859 (PMC3144958; doi:10.1371/journal.pone.0022859)
Supplement: Figure S2 — Comparison of enriched functions common to HK gene lists. This graph shows enriched functions common to most HK gene lists presented in Table 2. Functions enriched in at least eight out of nine HK lists are shown. The color reflects the negative logarithm-transformed FDR-adjusted EASE scores (see [53]). (PDF) [file pone.0022859.s002.pdf]

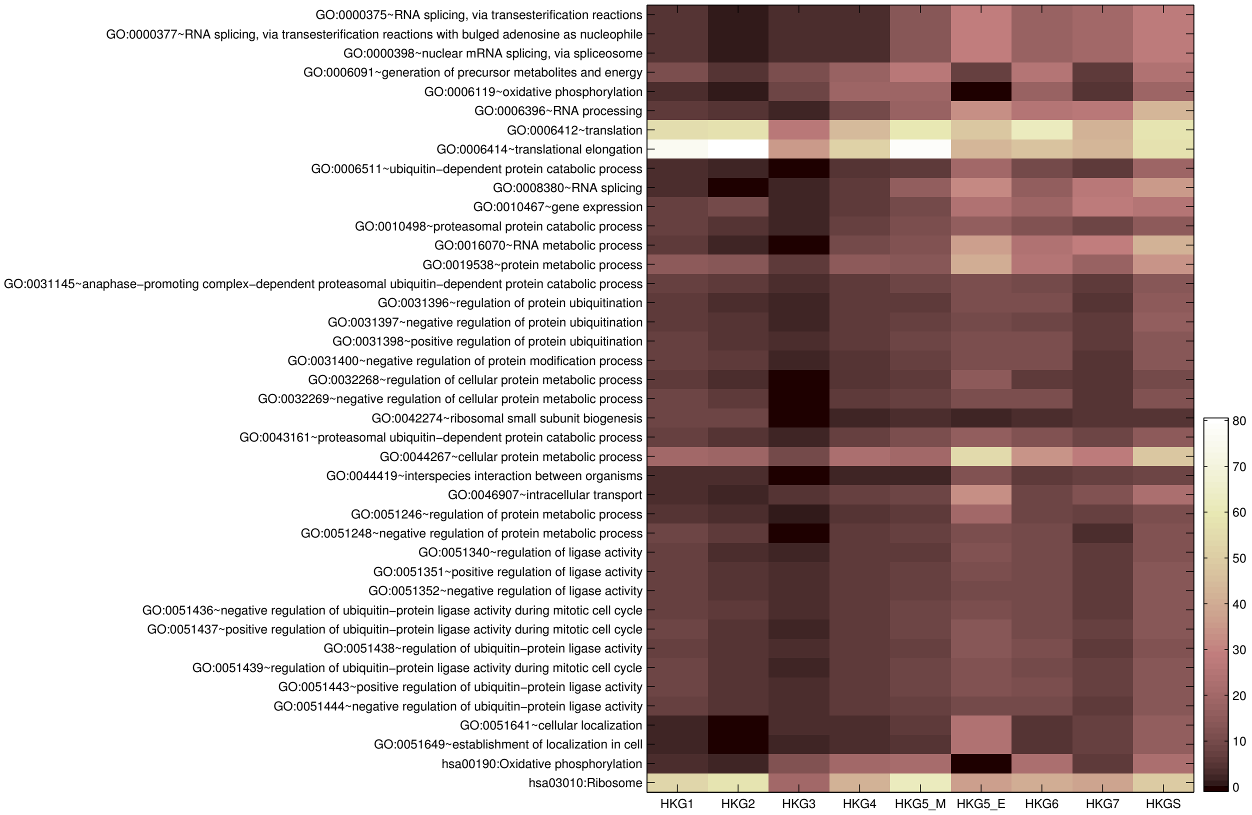

Figure S2

Comparison of enriched functions common to HK gene lists. This graph shows enriched functions common to most HK gene lists presented in Table 2. Functions enriched in at least eight out of nine HK lists are shown. The color reflects the negative logarithm-transformed FDR-adjusted EASE scores (see [53]).
