# Supplementary material for: Identification of Human Housekeeping Genes and Tissue-Selective Genes by Microarray Meta-Analysis
Source: PLoS One. 2011 Jul 27;6(7):e22859. doi: 10.1371/journal.pone.0022859 (PMC3144958; doi:10.1371/journal.pone.0022859)
Supplement: Figure S3 — Comparison of enriched functions unique to each HK gene list. This graph shows enriched functions unique to each HK gene list presented in Table 2. The color reflects the negative logarithm-transformed FDR-adjusted EASE scores (see [53]). (PDF) [file pone.0022859.s003.pdf]

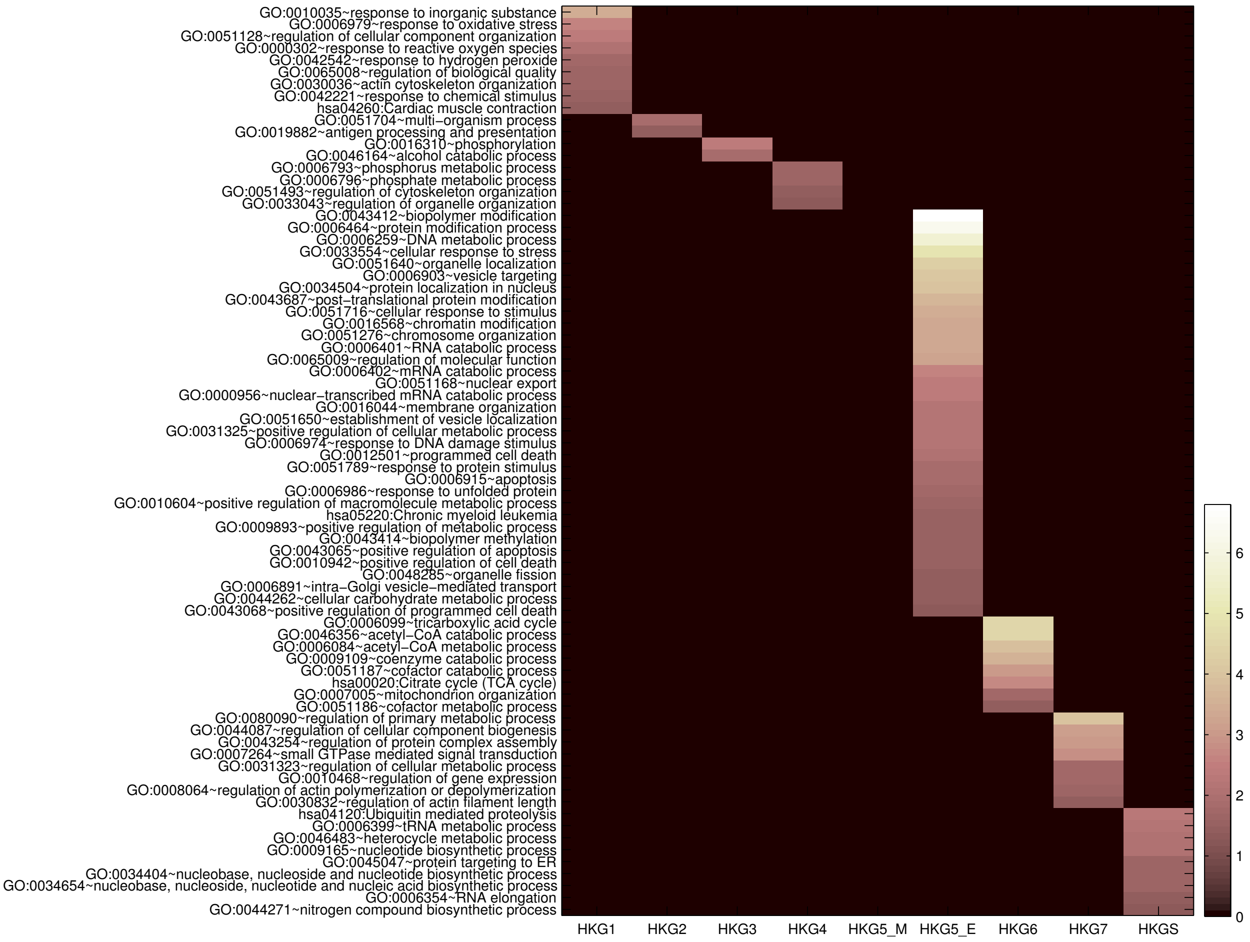

Figure S3

Comparison of enriched functions unique to each HK gene list. This graph shows enriched functions unique to each HK gene list presented in Table 2. The color reflects the negative logarithm-transformed FDR-adjusted EASE scores (see [53]).
