# Supplementary material for: Identification of Human Housekeeping Genes and Tissue-Selective Genes by Microarray Meta-Analysis
Source: PLoS One. 2011 Jul 27;6(7):e22859. doi: 10.1371/journal.pone.0022859 (PMC3144958; doi:10.1371/journal.pone.0022859)
Supplement: Figure S4 — Clustering of 43 tissues with expression pattern of TS genes. Tissues were hierarchically clustered with average linkage according to gene expression patterns (shown in Figure 3). Tissues are grouped with functional similarities. (PDF) [file pone.0022859.s004.pdf]

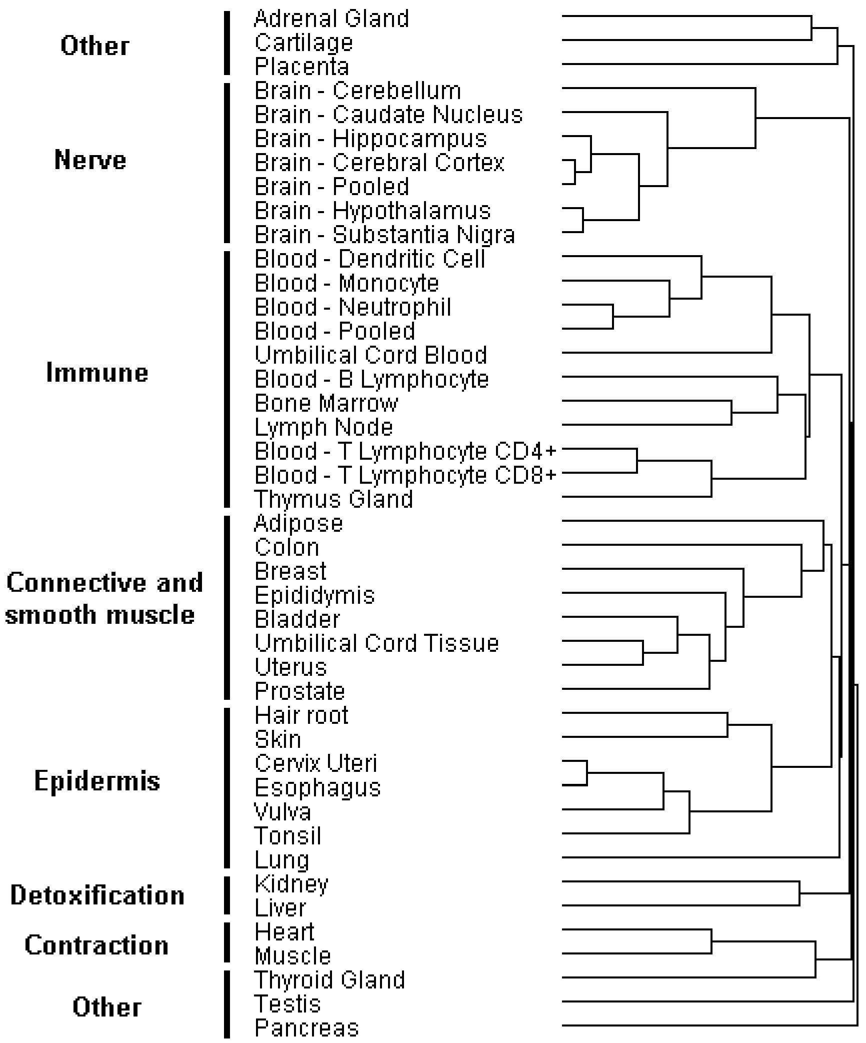

Figure S4

Clustering of 43 tissues with expression pattern of TS genes. Tissues were hierarchically clustered with average linkage according to gene expression patterns (shown in Figure 3). Tissues are grouped with functional similarities.
