# Supplementary material for: Identification of Human Housekeeping Genes and Tissue-Selective Genes by Microarray Meta-Analysis
Source: PLoS One. 2011 Jul 27;6(7):e22859. doi: 10.1371/journal.pone.0022859 (PMC3144958; doi:10.1371/journal.pone.0022859)
Supplement: Figure S5 — Percentage of expressed genes in each tissue with three different indicators. Percentages of expressed genes range from 36% (thyroid gland) to 55% (epididymis) by applying a cutoff of 100 to FPEI (green bars). These numbers are proportional to that identified by cutoff of 50% to fraction Present (red bars) and the mean percent presents of each tissue. Applying a cutoff of 200 (the scaling target of the MAS5 normalized data) to expression intensity tends to set 50% of genes as expressed (blue bars). Mean percent presents are not shown for clarity. (PDF) [file pone.0022859.s005.pdf]

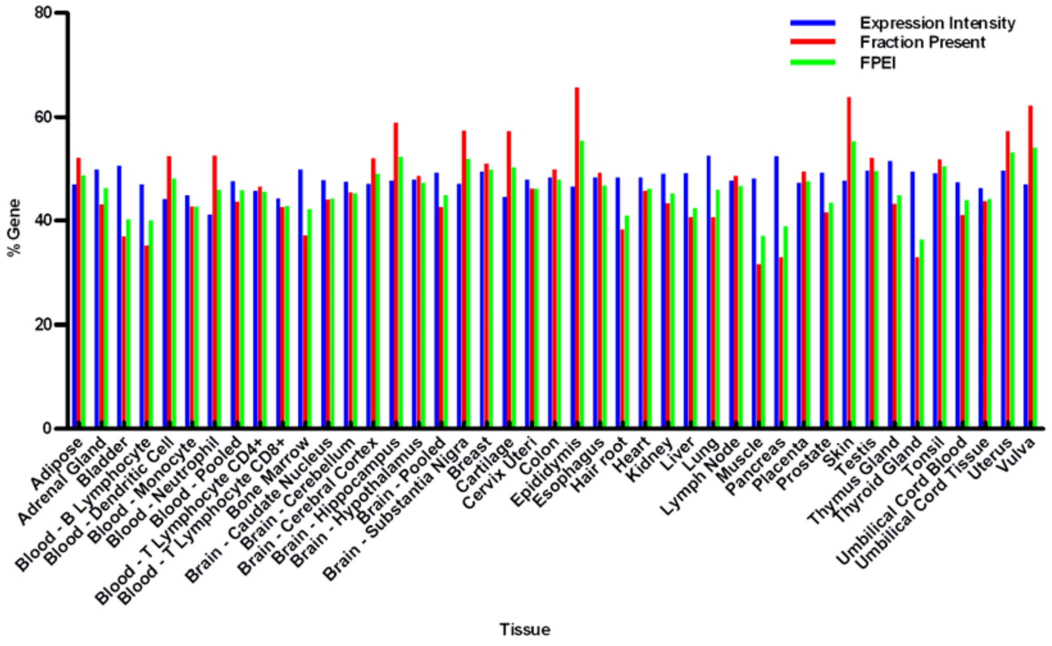

Figure S5

Percentage of expressed genes in each tissue with three different indicators. Percentages of expressed genes range from 36% (thyroid gland) to 55% (epididymis) by applying a cutoff of 100 to FPEI (green bars). These numbers are proportional to that identified by cutoff of 50% to fraction Present (red bars) and the mean percent presents of each tissue. Applying a cutoff of 200 (the scaling target of the MAS5 normalized data) to expression intensity tends to set 50% of genes as expressed (blue bars). Mean percent presents are not shown for clarity.
