# Supplementary material for: Identification of Human Housekeeping Genes and Tissue-Selective Genes by Microarray Meta-Analysis
Source: PLoS One. 2011 Jul 27;6(7):e22859. doi: 10.1371/journal.pone.0022859 (PMC3144958; doi:10.1371/journal.pone.0022859)
Supplement: Figure S6 — Comparison of percent present of samples in Gene Expression Atlas II to other experiments. Samples of Gene Expression Atlas II show a significant lower percent present when compared to other samples used in this study (P = 1.8×10^−64^ by two-tailed t-test). Bars are means from different sample origins; error bars show s.d.. (PDF) [file pone.0022859.s006.pdf]

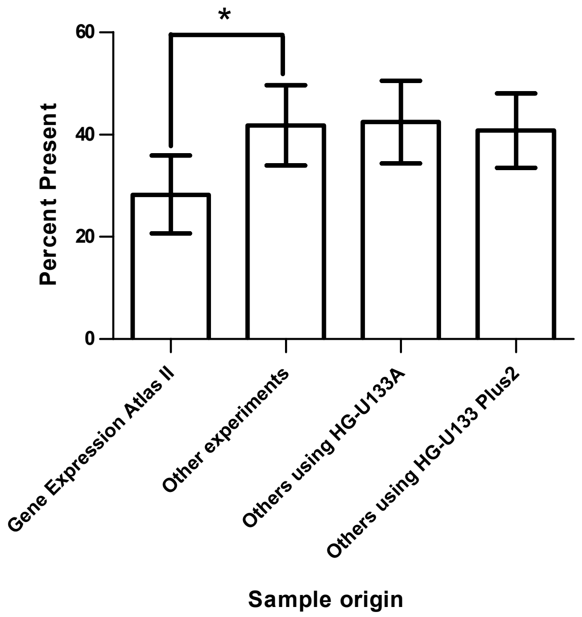

Figure S6

Comparison of percent present of samples in Gene Expression Atlas II to other experiments. Samples of Gene Expression Atlas II show a significant lower percent present when compared to other samples used in this study ($P = 1.8 \times 10^{-64}$ by two-tailed *t*-test). Bars are means from different sample origins; error bars show s.d..
