# Supplementary material for: Identification of Human Housekeeping Genes and Tissue-Selective Genes by Microarray Meta-Analysis
Source: PLoS One. 2011 Jul 27;6(7):e22859. doi: 10.1371/journal.pone.0022859 (PMC3144958; doi:10.1371/journal.pone.0022859)
Supplement: Figure S7 — Expression of seven both housekeeping and tissue-selective genes. FDX1 is selectively expressed in adrenal gland (2), and TUBA3C is selectively expressed in testis (36). Other five genes, BNIP3L, RNF10, MKRN1, ADIPOR1, and MARCH8, are selectively expressed in umbilical cord blood (40). (PDF) [file pone.0022859.s007.pdf]

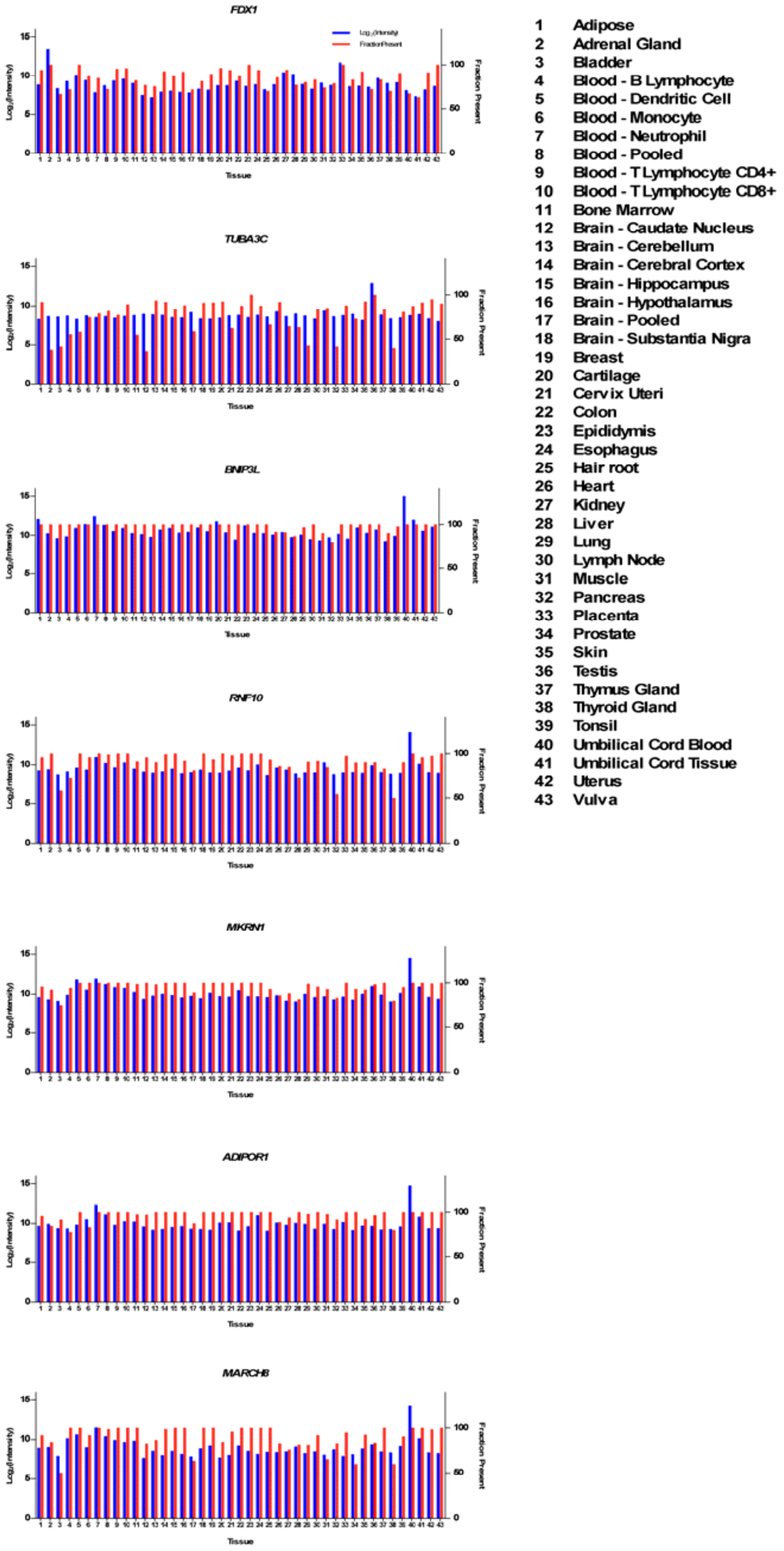

Figure S7

Expression of seven both housekeeping and tissue-selective genes. *FDX1* is selectively expressed in adrenal gland (2), and *TUBA3C* is selectively expressed in testis (36). Other five genes, *BNIP3L*, *RNF10*, *MKRN1*, *ADIPOR1*, and *MARCH8*, are selectively expressed in umbilical cord blood (40).
