# Supplementary material for: Identification of Human Housekeeping Genes and Tissue-Selective Genes by Microarray Meta-Analysis
Source: PLoS One. 2011 Jul 27;6(7):e22859. doi: 10.1371/journal.pone.0022859 (PMC3144958; doi:10.1371/journal.pone.0022859)
Supplement: Text S1 — Results for Receiver Operating Characteristic (ROC) analysis with 93 muscle samples. (PDF) [file pone.0022859.s013.pdf]

Text S1 Results for Receiver Operating Characteristic (ROC) analysis with 93 muscle samples.

To evaluate the three indicators and sample size in identifying representative protein-encoding transcriptomes, we performed the Receiver Operating Characteristic (ROC) analysis with 93 muscle samples. We chose muscle as an example due to their large amount of available samples and relatively uniform histological composition. We set target genes (that is, genes expected to be expressed in muscle) and background genes as genes in muscle contraction (GO: 0006936) and in other five non-muscular functions, respectively (GO: 0007417, central nervous system development; GO: 0019953, sexual reproduction; GO: 0050817, coagulation; GO: 0007586, digestion; GO: 0006955, immune response). This provided 127 target genes and 1,359 background genes with genes in intersections removed. Three indicators, expression intensity, fraction Present, and fraction Present weighted expression intensity (FPEI), were recalculated according to 1 to 100 samples randomly selected from the 93 muscle samples (with replacement). With a series of cutoffs for the recalculated indicators, the true positive rate (TPR) and false positive rate (FPR) were identified by the cutoff-passed target and background genes, respectively. The ROC curves were plotted with TPRs against FPRs, and the areas under the ROC curves (AUC) were calculated as the single-value-evaluation of the indicators. The procedure from random sampling to AUC calculation was iterated 100 times, and the results were then averaged. Figure in this Text represents the ROC curve of 100 randomly selected samples, and Figure 1 in the main text represents the AUCs with different numbers of randomly selected samples.

For all of the three indicators, results of the ROC analysis show that more than ten samples are required to reach a robust assessment of protein-encoding transcriptome of a tissue (Figure 1 in the main text). Additionally, as the ROC curves show, the FPEI inherited advantages from the other two indicators and overall performs better (Text S1 Figure). The expression intensity performed better with a high value when compared to fraction Present (the region from 0 to 0.3 of the FPR, which corresponded to an expression intensity higher than 400 or a fraction Present higher than 75%), but worse with a low value (the region from 0.3 to 0.8 of the FPR, which corresponded to expression intensity in a range of 50 to 400 or fraction Present in a range of 5% to 75%). This reflects the high-noise nature of low expression intensity. Weighting expression intensity with fraction Present has compensated this drawback while retaining the discriminability of high expression intensity, which caused the FPEI to outperform the others. Thus, we applied the FPEI to identify the representative protein-encoding transcriptomes for subsequent HK and TS genes exploration.

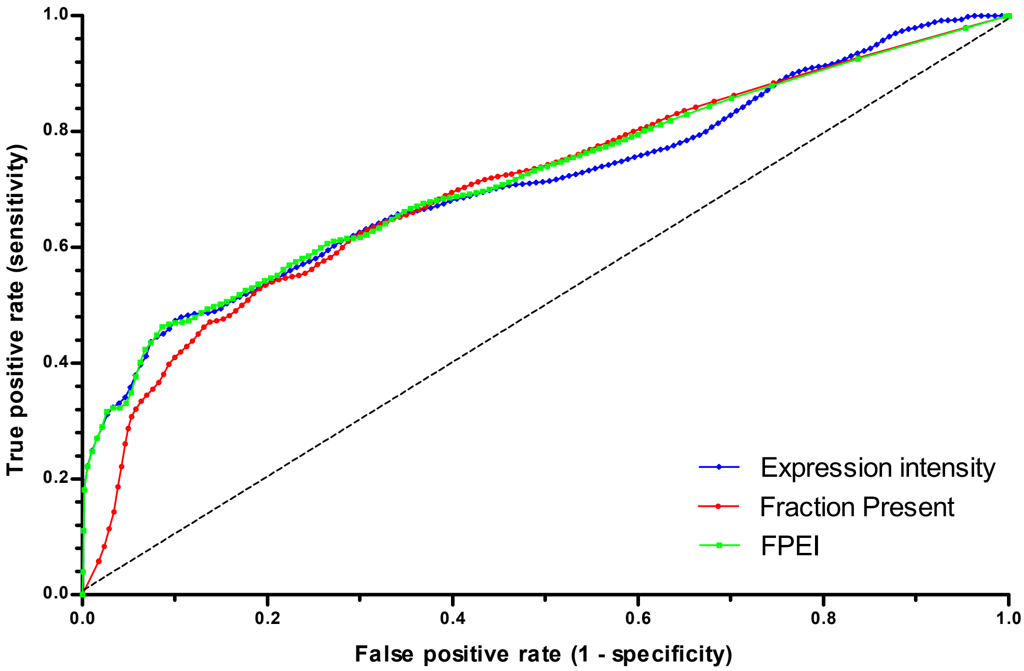

Text S1 Figure

Receiver Operating Characteristic (ROC) curves of 100 randomly selected muscle samples represent the characteristics of three indicators in selecting muscle contraction genes (GO: 0006936) from genes in other five non-muscular functions. The diagonal dashed line represents random guess line.
